# Developing Nutrition Label Reading Skills: A Web-Based Practice Approach

**DOI:** 10.2196/jmir.6583

**Published:** 2017-01-13

**Authors:** Lisa M Soederberg Miller, Laurel A Beckett, Jacqueline J Bergman, Machelle D Wilson, Elizabeth A Applegate, Tanja N Gibson

**Affiliations:** ^1^ Department of Human Ecology University of California, Davis Davis, CA United States; ^2^ Department of Public Health Sciences University of California, Davis Davis, CA United States; ^3^ Department of Nutrition University of California, Davis Davis, CA United States

**Keywords:** nutrition labeling, dietary habits, automatic information processing, food selection, choice behavior

## Abstract

**Background:**

Nutrition labels offer the information needed to follow Dietary Guidelines for Americans, yet many individuals use labels infrequently or ineffectively due to limited comprehension and the effort required to use them.

**Objective:**

The objective of our study was to develop and test a Web-based label-reading training tool to improve individuals’ ability to use labels to select more healthful foods. We were particularly interested in determining whether practice can lead to increased accuracy using labels as well as decreased effort, together reflecting greater efficiency. We compared a basic and an enhanced, prior-knowledge version of the tool that contained an additional component, a brief nutrition tutorial.

**Methods:**

Participants were 140 college students with an average age of 20.7 (SD 2.1) years and education 14.6 (SD 1.2) years, who completed 3 sets of practice that were designed to teach them, through repetition and feedback, how to use nutrition labels to select more healthful products. Prior to training, participants in the prior-knowledge group viewed a multimedia nutrition presentation, which those in the basic group did not receive. Mixed-effects models tested for improvement in accuracy and speed with practice, and whether improvements varied by group.

**Results:**

The training led to significant increases in average accuracy across the 3 practice sets (averaging 79% [19/24 questions], 92% [22/24], 96% [23/24] respectively, *P*<.001), as well as decreases in time to complete with mean (SD) values of 8.7 (2.8), 4.6 (1.8), and 4.1 (1.7) seconds, respectively. In block 3, the odds of a correct answer for the prior-knowledge group were 79% higher (odds ratio, OR=1.79, 95% CI 1.1-2.9) than those for the basic group (*P*=.02). There was no significant difference between the groups in block 2 (*P*=.89).

**Conclusions:**

Practice led to improvements in nutrition label reading skills that are indicative of early stages of automatic processing. To the extent that automatic processes are at the core of healthy habit change, this may be an efficient way to improve dietary decision-making.

## Introduction

The Dietary Guidelines for Americans are designed to support eating patterns that promote health and reduce the risk of diet-related chronic diseases [1,2]. The guidelines also call for manufacturers to include the Nutrition Facts panel (ie, nutrition label) on food labels to help individuals make informed choices. Deciding which foods are healthy when shopping for oneself or one’s family can be a daunting task, particularly when simultaneously factoring in price and convenience. Thus, it is not surprising that grocery shopping is considered to be the least enjoyable (tied with house cleaning) of 28 daily activities [3]. Although nutrition labels can facilitate decision-making, many individuals do not use nutrition labels effectively or at all [4-6]. Underutilization may be due to a lack of understanding of the information as well as an unwillingness to invest the time to understand them, which renders labels too effortful to use [7,8]. For example, individuals often have difficulty understanding the numerical information [9] and what amounts constitute a meaningful difference in calories or other nutrients when comparing products [10]. These challenges can lead individuals to avoid using nutrition labels.

Although many agree that more work is needed to increase education related to label use [5,6,11], there is no clear consensus on teaching individuals how to use nutrition labels, resulting in a wide range of approaches. Label reading tasks have been incorporated into a larger set of nutrition education goals in face-to-face delivery mode [12,13]. Using a randomized pretest-posttest design, for example, older women (n=98) with diabetes completed a series (10) of weekly sessions on nutrition, some of which included demonstrations on how to compare food labels [14]. The intervention led to increases in nutrition knowledge, self-efficacy, and label reading accuracy. Another approach has been to focus more directly on label reading. For example, researchers conducted a pretest-posttest intervention on food label reading across 9 weeks (n=43 women) and found greater gains on label reading outcomes in the intervention than in the control group [13]. In a single training session, consumers (n=19) attended a 2-hour lecture at a grocery store, followed by reading label practice [15]. The results showed improvements in self-reported knowledge and confidence using labels; however, improvements in label reading abilities were not reported. However, other studies using a small-group format within a single training session have reported improvements in label comprehension [16,17]. Evidence linking label reading training with dietary intake is scarce, but a 10- to 15-minute training session on how to read nutrition labels was insufficient to affect sodium intake 3 months later (label reading ability was not assessed) [18].

In addition to face-to-face nutrition interventions, there has been a rapid increase in the number of Web-based interventions [19], which have the advantage of delivering relatively low-cost, interactive nutrition interventions to a large number of consumers [19-21].

Some evidence suggests that individuals value Web-based educational tools as a way to guide food choices in the long run [22]. Web-based programs have shown success in improving the understanding of nutrition as well as dietary quality [19,23], even though they do not consistently surpass other methods [24,25]. However, we are aware of only a few studies that have examined label reading skills [26,27]. In one study that compared face-to-face and Web-based training, researchers taught participants 3 nutrition topics, 1 of which was label reading [26]. The results showed that participants in the face-to-face training showed greater improvements in label reading accuracy than those in the Web-based training. However, for the other 2 topics, both delivery formats improved nutrition-related behaviors to the same extent. Specific details surrounding the nature of label reading activities (eg, focus and process) were not provided; however, the researchers suggested that label reading may be relatively more difficult and therefore participants may benefit more from in-person education delivery [26]. In this study, we examined the impact of a Web-based label reading tool to increase label reading accuracy and decrease time to read labels. The tool was specifically designed to promote skill acquisition [28] through deliberate practice [29] and to promote efficiency, that is, increase accuracy and reduce time due to greater automaticity. Automatic processes are desirable because they are relatively fast and effortless compared with more controlled processes [30,31] and can be developed through intense practice, or repetition [29,32,33] using tasks that provide meaningful content and feedback [34].

We examined a basic version of the training as well as an enhanced version that provided prior nutrition information, in the form of self-paced slides. Past work has shown that prior knowledge supports the acquisition of new knowledge and skills [35], and correlational studies have shown associations between prior nutrition knowledge and label comprehension [36]. Thus, participants were assigned to the basic group or an enhanced, prior-knowledge group to determine whether label reading accuracy increased, and time to read labels decreased, with practice and whether prior knowledge affected practice effects.

## Methods

### Recruitment

Participants were 140 college students with an average age of 20.7 (SD 2.1) years and education 14.6 (SD 1.2) years, who were enrolled in 1 of 2 consecutive quarters of an introductory psychology course. Recruitment occurred through a Web-based portal that listed the following eligibility criteria: ability to read from a computer screen and use a computer mouse, and English language fluency. To balance the number of participants within groups, assignment occurred through a Web-based system by alternating between the 2 conditions at sign-up, with the first participant each quarter randomly assigned to a group. Ethical approval was obtained from the Institutional Review Board at the University of California, Davis.

### Measures of Demographics and Prior Food Label Experience

Participants completed a demographic survey, followed by measures assessing prior food label experience including self-reported food label use and nutrition label numeracy. Self-reported food label use was assessed using the question: “I’d like you to think about the labels on many food products that list ingredients and provide nutrition and other information. When you buy a product for the first time, how often do you read this information?” [37]. Responses were made on a scale of 1 (never) to 5 (always). Nutrition label numeracy was assessed using multiple-choice questions (n=7) requiring participants to manipulate quantitative information, for example, “Roughly how many servings of this product would you need to get 100% of the recommended daily value of iron?” [38,39]. Scores were the total number of questions answered correctly.

### Enhancement (Prior-Knowledge) Manipulation

Those in the prior-knowledge group received a 7-minute nutrition overview using PowerPoint slides that taught basic nutrition information (eg, definition of nutrients and energy, sources of nutrients, diet-health relations). Those in the basic group did not receive the nutrition overview. Both groups were then given a multiple-choice nutrition knowledge quiz (18 items), designed to assess learning from the overview for the prior-knowledge group compared with the basic group.

### Nutrition Label Training Task

Two of the most common reasons for using nutrition labels are to compare foods and select healthful options [40]; however, individuals lack awareness of what constitutes an important nutrient difference between 2 products [7,10]. For example, individuals who were asked to compare 2 similar products paid attention to nutrients that differed insignificantly across the products or were not particularly salient for the food type, for example, paying attention to sodium levels in cold cereals while ignoring added sugar and fiber [10]. Thus, the task we designed to develop label-reading skills provided an opportunity to identify meaningful nutrient differences that signify relative healthfulness of a variety of foods (eg, cereals, and soups). We included to-be-limited as well as to-be-encouraged nutrients, which are often overlooked [6]. Because nutrition label reading is a complex skill, we targeted the underlying processes supporting healthfulness decisions and removed other food label components (eg, pictures of food, claims) that could potentially slow the early stages of skill acquisition.

**Figure 1:**
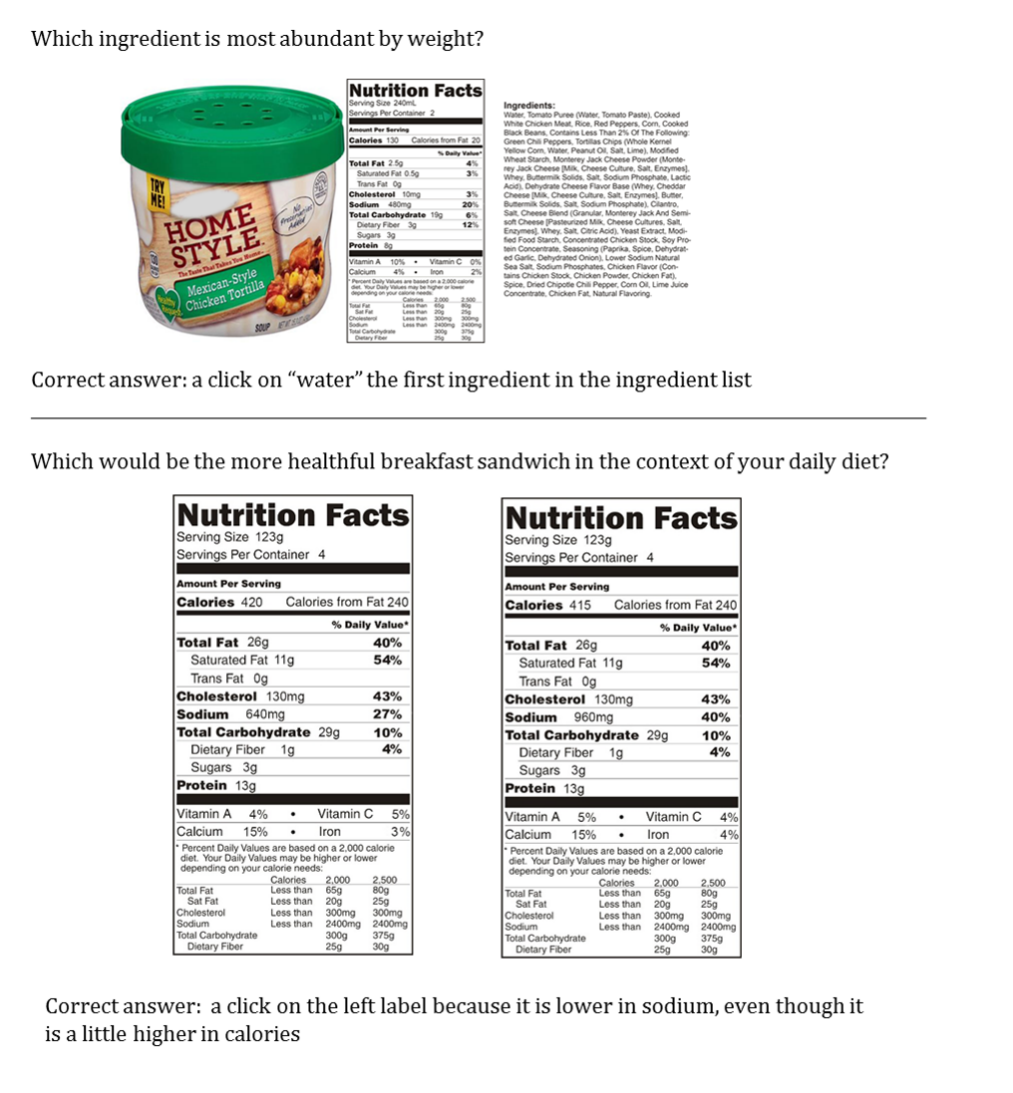
Samples of training materials for locate task (top) and comparison task (bottom).

The nutrition labels for the training task were based on actual foods from different meal types (breakfast, lunch, dinner, snacks, and beverages) and included examples of more and less healthful foods within each meal type (eg, potato chips, carrot sticks, brown rice vegetable bowl, fried fish sandwich). From these foods, we manipulated the nutrition information to create 200 pairs for the comparison task. Correct answers were operationalized in terms of large nutrient differences between pairs, referred to as *large-consistent* differences. We also manipulated small differences, which were made in the opposite direction, and thus referred to as *small-inconsistent* differences. Finally, in addition to the size of the difference, we manipulated the type of nutrient to include micronutrient only (eg, calcium), to-be-encouraged only (eg, fiber), to-be-encouraged and to-be-limited (eg, fiber and sodium), and to-be-limited only (eg, sodium) differences. In general, we expected greater accuracy for pairs with large-consistent to-be-limited nutrient differences relative to to-be-encouraged nutrient differences. On the contrary, pairs with only small-inconsistent to-be-encouraged differences would be relatively easier than the others because this information would be less likely to be used [6], and therefore less likely to mislead individuals. We included 3 introductory label tasks prior to the focused practice comparing nutrition labels. The first task described the information available on a food label (eg, the different types of nutrients, metric types). The second task (Figure 1) required participants to locate a specific piece of information on 1 of 3 areas of the food label (nutrition label, ingredient list, or front of package). The third task consisted of a set of 4 sample comparisons tasks, followed by the correct answer and answers to any follow-up questions.

The label reading training task provided practice using nutrition labels (pre-2018 format) to compare the relative healthfulness of pairs of foods. Participants completed 3 blocks, each containing 24 nutrition label comparisons, followed by feedback after each comparison (correct/incorrect) as well as their percent correct at the end of each block. For each comparison, 2 nutrition labels were presented side by side, with instructions to select the label that represented the more healthful option within the context of their daily diet (Figure 1). The location of the more healthful product was counterbalanced across the left and right sides of the screen. At the end of the training, participants rated their perceptions of the training task on a 5-point scale. The tasks were completed in roughly 60-90 minutes.

### Perceptions of Training

We obtained feedback from participants regarding their experience with the training tool, drawing on past research on Web-based training [41]. We assessed participants’ perceptions of the label comparison task to determine whether they felt they had increased their accuracy and decreased their time to respond with practice, and whether they felt their skills would improve with more practice. We used 2 items: “Regarding nutrition label comparisons you just completed, to what extent did they help you: i) compare foods more accurately, ii) compare foods more quickly?” and responses were made on a 5-point scale: 5=very helpful; 1=not very helpful. In addition, we asked 2 questions about the entire session to assess perceptions of overall utility: “Regarding the entire session, to what extent were the tasks: i) easy to complete, ii) useful to you?” We also asked participants whether they felt they would improve their food label reading skills with additional practice. Responses were made on a 5-point scale.

### Statistical Analyses

All statistical analyses were performed using SAS software version 9.4 (SAS Institute). We tested for differences between prior-knowledge and basic groups in age, food label use, numeracy, and education using the Wilcoxon rank-sum test, as well as sex and Hispanic ethnicity using the chi-square test.

### Accuracy

At the comparison level (accuracy=correct or incorrect for each comparison), we fitted a mixed-effects hierarchical logistic regression model to test for the effects of prior knowledge on training improvement, controlling for sex, self-reported food label use, numeracy, and comparison type (ie, large-consistent versus small-inconsistent differences for each pair). Quarter enrolled was also included to control for possible differences between the 2 samples. We tested for interactions between training block and prior-knowledge group to test for the effects of knowledge on training improvement. We also tested for interactions between training block and comparison type to determine where the benefits of training were the most evident. Bivariate associations were also tested for each covariate using mixed-effects logistic regression models, so that both raw and adjusted odds ratios (OR) could be obtained.

### Time to Complete Blocks

In addition to accuracy, we explored the amount of time individuals took to complete blocks to determine whether there was evidence for initial stages of automaticity. Block *duration* was defined as the average time to complete each block. Duration was log10-transformed to achieve normality of the residuals and analyzed using mixed-effects regression models that tested for a downward trend in duration with block. We also tested for the effects of sex, quarter enrolled, self-reported food label use, numeracy, and prior-knowledge group and its interaction with block to test for whether prior knowledge affected duration with practice. Bivariate associations were also tested for each covariate, so that both raw and adjusted effects could be assessed.

## Results

### Enrollment

The initial enrollment included 151 participants; 11 (8 from the prior-knowledge group and 3 from the basic group) were eliminated because their very low accuracy (at or below the chance performance level of 50%) indicated that they were unable or unwilling to follow instructions. The characteristics of the remaining 140 participants by group are shown in Table 1.

**Table 1:** Bivariate associations of baseline characteristics with treatment group.

Effect (units)	Estimates (SD or SE)^a^		*P* value
	Prior-knowledge (n=67)	Basic (n=73)	
Age (years)	20.5 (2.1)	20.9 (2.2)	.31
Education (years)	14.4 (1.3)	14.7 (1.2)	.18
Numeracy (number correct out of 7)	4.5 (1.9)	4.5 (2.1)	.86
Self-reported food label use (Likert scale, 1-5)	3.5 (1.1)	3.9 (1.1)	.04
Female	60% (4.4)	60% (4.6)	.92
Hispanic	16% (4.6)	16% (4.4)	.99

^a^Estimates are mean (SD) except for “Female” and “Hispanic,” which are in percent (SE).

There was a significant difference between the groups in their self-reported food label use, with those in the basic group reporting slightly higher label use than those in the prior-knowledge group. There were no significant differences in age, numeracy, or education. As a manipulation check, we examined the effects of prior-knowledge group on nutrition quiz scores to determine whether the overview of nutrition increased nutrition knowledge. There was a significant difference between groups on the nutrition quiz such that the prior-knowledge group had an average of 64% correct and the basic group had an average of 51% correct (*P*<.001). Numeracy had a significant effect on the average score (*P*<.001), but there were no significant differences in quiz score due to sex, quarter, or self-reported food label use (*P*=.66, .26, and .14, respectively).

### Accuracy

The effects of predictors on accuracy (odds of answering a comparison correctly) are shown in Table 2 as univariate unadjusted and model-adjusted ORs, CIs, and *P* values. Both the adjusted and unadjusted analyses on accuracy showed that on average individuals increased markedly in their accuracy with practice. Accuracy increased on average from 79% in the first block to 92% and 96% in the second and third blocks, respectively (*P*<.001) in the bivariate association. In the full model, the interaction between prior knowledge and block was significant (*P*=.02). In block 3, the odds of a correct answer for the prior-knowledge group were 79% higher than those for the basic group (*P*=.02). There was no significant difference between the groups in block 2 (*P*=.89).

Accuracy depended on the type of comparison. As expected, accuracy was higher for pairs with to-be-limited large-consistent differences relative to other large-consistent differences (micronutrients, to-be-encouraged only, both to-be-encouraged and to-be-limited) (*P*<.001). To illustrate, in block 1, predicted accuracy was 80% for the to-be-limited large-consistent differences compared with 53% for the micronutrient large-consistent differences. On the contrary, accuracy was the lowest for the combined to-be-encouraged and to-be-limited small-inconsistent differences (relative to micronutrients or to-be-encouraged only) types (*P*<.001). For example, in block 1, participants had a predicted accuracy of only 78% for the combined small-inconsistent differences, but 86% for the micronutrient small-inconsistent differences.

**Figure 2:**
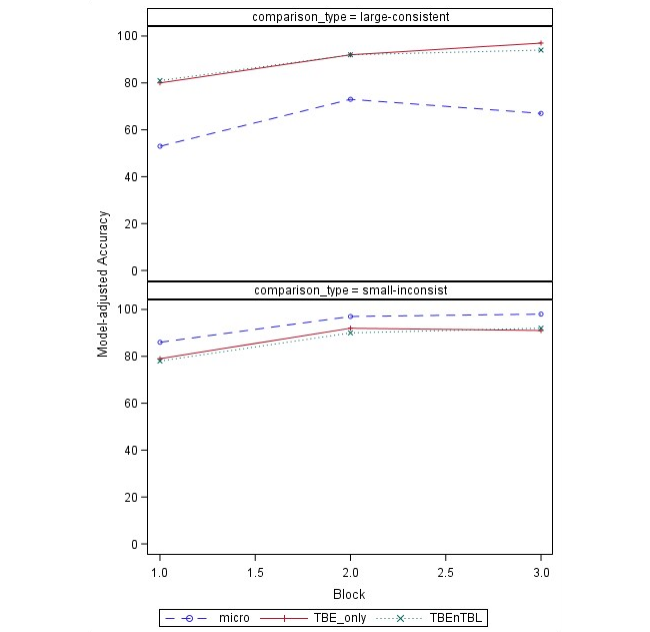
Model-adjusted accuracy for large-consistent (top) and small-inconsistent (bottom) nutrient differences by block. Micro=micronutrients; TBE: to be encouraged; TBL: to be limited.

The magnitude of improvement also varied by comparison type, as reflected in significant interactions between training block and small-inconsistent differences (*P*<.001), and between training block and large-consistent differences (*P*<.001). Pairs with to-be-limited large-consistent differences showed the highest proportion of correct responses but were similar to pairs with to-be-encouraged and combined to-be-encouraged and to-be-limited differences. Accuracy for the micronutrient large-consistent differences remained significantly lower across blocks. In contrast, the pairs with micronutrient small-inconsistent differences had the highest proportion of correct responses across all blocks, while the pairs with to-be-encouraged and to-be-limited small-inconsistent differences had lower and similar levels of accuracy. Figure 2 shows average, model-adjusted accuracy differences across comparison types, by block.

Numeracy significantly predicted accuracy (*P*<.001), such that, for every unit increase in numeracy, the odds of a correct response increased by 11%. However, gender (*P*=.06), self-reported food label use (*P*=.40), and quarter enrolled (*P*=.12) all showed nonsignificant effects.

**Table 2:** Effects on accuracy, univariate unadjusted (raw) and adjusted.

Effect			Unadjusted (raw)			Adjusted		
			Odds ratio	95% CI	*P* value	Odds ratio	95% CI	*P* value
**Manipulation**								
	Prior-knowledge group (reference=basic)		1.03	.77-1.4	.85	1.03	0.76-1.4	.84
	Block 3		5.74	4.7-7.1	<.001	4.2	1.6-10.9	.003
	2		2.86	2.4-3.4		2.1	0.79-5.7	.14
	1		1	-		1	-	-
	Sm^a^ inconsistent differences TBE^b^ and TBL^c^		.30	.25-.37	<.001	.45	0.33-0.61	<.001
	TBE only		.43	.32-.58		.58	0.37-0.90	.01
	Micro^d^		1	-		1	-	-
	Lg^e^ consistent differences TBE and TBL		2.9	1.9-4.3	<.001	2.18	1.12-4.22	.02
	TBE only		2.9	2.0-4.2		2.47	1.2-5.0	.13
	TBL only		4.8	3.3-7.2		3.23	1.6-6.3	<.001
	Micro		1	-		1	-	-
**Interactions**								
	*Block*	*Prior-knowledge group*						
	3	Knowledge				1.79	1.1-2.9	.02
	2	Knowledge				.97	0.66-1.4	.89
	(ref) 1	Basic				-	-	-
	*Block*	*Sm Inconsistent*						
	3	TBE and TBL				.42	0.23-0.78	.006
	3	TBE only				.17	0.08-0.36	<.001
	2	TBE and TBL				.60	0.33-0.93	.03
	2	TBE only				.37	0.17-0.78	.009
	(ref) 1	Micro				-	-	-
	*Block*	*Lg Consistent*						
	3	TBE only				4.6	2.1-10.6	<.001
	3	TBL only				18.2	6.1-58.8	<.001
	3	TBE and TBL				10.8	4.2-29.0	<.001
	2	TBE only				1.34	0.58-3.1	.49
	2	TBL only				1.80	0.78-4.7	.21
	2	TBE and TBL				1.22	0.46-2.9	.67
	(ref) 1	Micro				-	-	-
**Participant characteristics**								
	Sex (ref=female)		1.4	1.04-1.9	.03	1.55	1.1-2.2	.01
	Self-reported food label use		1.2	1.02-1.4	.02	1.04	0.89-1.21	.62
	Numeracy		1.2	1.1-1.3	<.001	1.11	1.01-1.21	.03
	Nutrition overview quiz		1.3	1.2-1.4	<.001	1.24	1.11-1.40	<.001
	Quarter enrolled (ref=2nd)		1.1	.81-1.5	.52	.77	0.55-1.07	.12

^a^Sm: Small.

^b^TBL: To be limited.

^c^TBE: To be encouraged.

^d^Micro: Micronutrients.

^e^Lg: Large.

**Table 3:** Time (log duration) to complete blocks with practice, raw and adjusted with back-transformed (exponentiated) coefficients.

Effect (reference level)	Raw coefficient	SE	Exponentiated coefficients	*P*	Adjusted coefficients	SE	Exponentiated coefficients	*P*
Block (ref) 1	-				-			
2	–0.28	0.009	0.53	<.001	–0.28	0.01	0.52	<.001
3	–0.34	0.009	0.46	<.001	–0.34	0.01	0.46	<.001
Prior-knowledge group (control)	0.015	0.023	1.04	.53	0.015	0.024	1.04	.54
Quarter enrolled (2nd)	0.011	0.024	1.03	.63	0.028	0.027	1.07	.27
Sex (female)	0.043	0.025	1.10	.67	0.052	0.026	1.13	.04
Self-reported food label use	0.006	0.011	1.01	.61	0.006	0.012	1.01	.65
Numeracy	–0.003	0.006	0.99	.60	–0.001	0.007	1.002	.85

### Time to Complete Blocks

As shown in Table 3, the speed at which individuals completed the blocks increased with practice. The mean duration of comparisons was 8.7 (SD 2.8) seconds for the first block, compared with 4.6 (SD 1.8) and 4.1 (SD 1.7) seconds for the second and third blocks (*P*<.001), respectively. Females, 6.0 (SD 3.1), took longer than males, 5.4 (SD 2.6), on average across blocks (*P*=.04). Numeracy (*P*=.85), self-reported food label use (*P*=.65), prior-knowledge group (*P*=.54), and quarter enrolled (*P*=.27) were all nonsignificant in the adjusted model.

### Perceptions of the Training

Data on perceptions of training were available for only 101 of the 140 participants due to a programming error. Within this subset, the majority of respondents rated the training positively, stating that the training was helpful or very helpful in comparing foods more accurately, 77% (78/101), and more quickly, 85% (86/101). Similarly, most participants indicated that the entire session was easy or very easy to complete, 79% (80/101), and was useful or very useful, 77% (78/101). Participants also felt that they were likely or very likely to improve their food label reading skills with additional practice, 76% (77/101).

## Discussion

### Principal Findings

There has been a paucity of research on nutrition label skill development. Drawing on the cognitive literature [29], we developed and tested a Web-based label training tool that focused heavily on practice. Participants were asked to make repeated nutrition label comparisons (72 in all) to learn what constitutes a meaningful difference between 2 products. After each comparison, we provided accuracy feedback so individuals could track their performance and adjust their approach. The findings showed significant improvements in label reading accuracy as well as decreased time to read the labels. Despite the high number of practice trials, participants viewed the training tasks as useful and easy to complete.

Most of the past research focusing on nutrition label training has been conducted face to face. These findings generally show that training increases label reading accuracy [16,17]. Research on label reading effects on dietary intake is scant, with 1 study showing no effects of training on sodium intake; however, the training lasted 10-15 minutes and no assessments of training efficacy were reported [18]. A handful of Web-based educational studies have been conducted, but details surrounding label tasks are often not reported [26,27]. For example, Park et al [27] used interactive, self-assessment quizzes with immediate feedback as part of a Web-based nutrition intervention for college students. One quiz topic focused on label reading; however, the nature of the task and the extent of improvement were not reported.

The results of this study shed light on the components of nutrition label training that may support long-term skill development. With practice, individuals were able to identify important or large nutrient differences between the 2 products without getting distracted by misleading minor nutrient differences (ie, those pointing them toward the less healthful option). Consistent with past research suggesting that to-be-encouraged nutrients are less likely to be considered [6], large-consistent to-be-encouraged differences were harder to evaluate but small-inconsistent to-be-encouraged differences were easier to ignore (less distracting). These findings build on earlier work showing that individuals are often unable to differentiate between insignificant and meaningful differences when comparing food products [10] and extend it by showing that the type of nutrient can exacerbate this problem. Importantly, individuals were able to identify these critical differences in nutrient levels with less time, suggesting that automatic processes supported skill development. Participants’ subjective experience of the training was consistent with these objective measures as reflected in perceptions of increased accuracy and speed.

The findings surrounding the prior-knowledge manipulation were consistent with the expectation that nutrition knowledge supports label reading skills [36]. Participants who received the nutrition overview showed significantly greater improvement across blocks relative to those in the basic condition. It is important to point out, however, that the effect of prior knowledge was small relative to the improvements across blocks (ie, the main effect of practice). Nevertheless, it was significant after controlling for past experience, as reflected in self-reported food label use (frequency) and numeracy skills. The additional nutrition knowledge likely supported comprehension of the various nutrients, daily values, and in turn what constitutes large versus small differences across products. It could also be that additional nutrition information motivates individuals to try to understand and learn new nutrition information [42]. Prior knowledge did not have a similar effect on increases in speed with practice. Although this was somewhat surprising, past work has shown that prior knowledge is sometimes more closely associated with accuracy than with speed [43].

The results of this study also indicate that the effects of prior knowledge, past experience, and numeracy on skill development differ. Specifically, self-reported frequency of use had little effect on accuracy; however, food label numeracy was significantly related to accuracy. This finding is consistent with research showing that self-reported frequency of label use is not necessarily indicative of how well food labels are understood [44].

The potential for nutrition labels to communicate nutrition information is widely acknowledged, as is the need for more experimental research to determine how the potential can be actualized [45]. Training for automaticity offers another avenue to increase nutrition awareness and improve dietary decision making. Although somewhat distinct from approaches based on habit theory, which sometimes downplay the role of nutrition education and information seeking [46], there is overlap. Both habit theory and the skill development approach in this study support the role of automaticity in behavior change. For some areas of behavior, skill development and knowledge acquisition may be necessary to establish the foundation of new habits, as well as the instigation and execution of habits within the complexities of daily life [47].

### Limitations and Future Directions

Although the sample size was small, it was consistent with other studies [18]. The sample was also young and well educated, making it unclear whether the findings generalize to middle-aged and older adults, and those with lower literacy and numeracy. College students are at risk of making poor dietary choices [48]; however, consumers with lower literacy and numeracy are also at risk [9] and require attention in future label training research. Another limitation was a ceiling effect on the last block, which might have prevented us from finding greater improvements on the last block. Interestingly, participants felt they would continue to improve their label reading skills if they were to continue to practice beyond the 3 blocks offered in the training session. Thus, despite the fact that performance was high, there was some understanding that skill development could continue with sustained practice. More work is needed to develop more challenging comparisons for educated consumers and examine dose-response training factors to better understand skill maintenance over time. Although we isolated the nutrition label from the rest of the package to allow individuals to focus on the most informationally dense part of the food label, the nutrition labels, future research is needed to examine nutrition label skill development as it occurs within the context of other parts of the package and in other settings (eg, Web-based or in-store grocery shopping).

### Conclusions

Consumers may not bother using nutrition labels if reading them is too difficult or time-consuming [49]. The findings of this study indicate that nutrition label-reading skills can become more efficient using a scalable, Web-based tool to provide focused practice. To the extent that continued practice will build automatic and efficient processes, additional training sessions may promote habitual and effective use of nutrition labels. Additional work is needed to determine whether efficient skills are more likely to be used when selecting foods to eat, and will in turn support the initiation and maintenance of healthful food choices in the long run.
